# Oxide Scale Microstructure and Scale Growth Kinetics of the Hot-Pressed SiBCN-Ti Ceramics Oxidized at 1500 °C

**DOI:** 10.3390/ma17133118

**Published:** 2024-06-25

**Authors:** Hao Peng, Haobo Jiang, Daxin Li, Zhihua Yang, Wenjiu Duan, Dechang Jia, Yu Zhou

**Affiliations:** 1 State Key Laboratory of Precision Welding & Joining of Materials and Structures, Harbin 150001, China; 2 Institute for Advanced Ceramics, School of Materials Science and Engineering, Harbin Institute of Technology, Harbin 150001, China; 3 Key Laboratory of Advanced Structural-Functional Integration Materials & Green Manufacturing Technology, Harbin Institute of Technology, Harbin 150001, China; 4 Tsinghua Shenzhen International Graduate School, Tsinghua University, Shenzhen 518055, China

**Keywords:** SiBCN-Ti, oxidation resistance, oxide layer, microstructural evolution

## Abstract

In this study, the SiBCN-Ti series ceramics with different Ti contents were fabricated, and the oxidation resistance and microstructural evolution of the ceramics at 1500 °C for different times were explored. The results show that with the increase in oxidation time, pores and bubbles are gradually formed in the oxide layer. When the oxidation time is less than or more than 4 h, the Ti(C, N) in the ceramics will maintain its initial structure or mostly transform to TiN. The introduction of Ti content can promote the formation of rutile silicate glass, thus healing the cracks and improving the oxidation resistance of the ceramics effectively.

## 1. Introduction

With the rapid development of aerospace technology, high-temperature structural ceramics have attracted increasing attention [1,2,3]. As a new type of high-temperature silicon-based non-oxide ceramics, SiBCN ceramics show great application potential in the aerospace industry due to their excellent oxidation, high-temperature, creep, and thermal shock resistance [4,5,6,7,8].

Normally, the SiBCN ceramics can be fabricated by precursor pyrolysis or mechanical alloying method [9,10,11,12]. However, SiBCN ceramics release a large amount of gas and show an obvious weight loss and volume shrinkage during high-temperature pyrolysis procedure, thus weakening the intrinsic mechanical properties and reducing the reliability of the ceramics [13,14]. Compared with the precursor pyrolysis method, the mechanical alloying method has obvious advantages, including being time-consuming, inexpensive, non-toxic, and easy to operate [15,16]. Additionally, the mechanical alloying approach is suitable for the preparation of large-size bulk ceramics [17,18].

In recent years, the microstructure, mechanical properties, thermophysical properties, and ablation resistance of SiBCN ceramics prepared by the mechanical alloying method have been systematically investigated in an in-depth study. A series of reinforcing phases, such as MWCNTs, graphene, carbon fibers, Mo, Al, and ZrB_2_, were adopted to optimize the properties of the SiBCN ceramics [19,20,21,22]. Among the above reinforcing phases, the introduction of ultra-high-temperature ceramics shows a great enhancement in the high-temperature properties of SiBCN ceramics. Miao et al. [23,24] prepared the SiBCN/ZrB_2_ composite ceramics by sol–gel method combined with spark plasma sintering, and the mechanical properties and ablation resistance of the composite ceramics exhibited obvious improvement. Nevertheless, after oxidation, the oxidation of ZrB_2_ provided a large amount of channels for the diffusion of oxygen, thereby decreasing the oxidation resistance of SiBCN ceramics [23]. Liao et al. [25] introduced TaSi_2_, HfSi_2_, and MoSi_2_ into SiBCN ceramics, and the oxidation test results showed that the proper content of the above ultra-high temperature ceramics could improve the oxidation resistance of SiBCN ceramics efficiently. Therefore, the introduction of ultra-high-temperature ceramics is a suitable strategy to improve both the high-temperature and anti-oxidation properties of SiBCN ceramics.

As a typical ultra-high-temperature ceramic, Ti(C, N) exhibits remarkable properties, including low density, high hardness, low coefficient of friction, good thermal and chemical stability, high melting point, high modulus of elasticity, and excellent thermodynamic stability [26,27,28]. Therefore, it has become the most commonly used metal carbide for the latest generation of metal-ceramic production. In our previous study [29], the Ti content was introduced into the SiBCN-Ti series ceramics by mechanical alloying method, and the Ti(C, N) phase was in situ formed during the hot-pressing procedure. The mechanical test results showed that the introduction of Ti content was prone to improvement in the bending strength and fracture toughness. Except for the mechanical properties, the anti-oxidation properties are also important to evaluate the application feasibility of SiBCN-based ceramics in extreme environments [30,31,32,33,34,35]. Up to now, the studies of the anti-oxidation properties of SiBCN-Ti ceramics are not sufficient, and the influential mechanism of the Ti content on the oxidation resistance of SiBCN ceramics is still not clear.

Therefore, in this study, the SiBCN-Ti series ceramics with different Ti contents were prepared by mechanical alloying plus hot-pressing sintering, and the anti-oxidation properties of the ceramics at 1500 °C for different times were investigated. The effects of the Ti content on the oxidation resistance and microstructural evolution were analyzed in depth.

## 2. Experimental Procedure

### 2.1. Preparation of SiBCN-Ti Powders

The SiBCN-Ti powders with different Ti contents were prepared by the mechanical alloying method [29]. The first mechanical alloying process was used to prepare TiN-TiB_2_ nanocrystalline powders. The Ti (particle size 15 μm, Ti content > 99.99%, Shanghai Maclean Biochemical Technology Co., Ltd., Shanghai, China) and h-BN (particle size 0.6 μm, BN content > 98%, Shandong Qingzhou Fangyuan Boron Nitride Plant, Qingzhou, China) mixed powders with a molar ratio of 3:2 were added into the ball milling tank containing Si_3_N_4_ balls, and the ratio of ball-to-powder was 20:1. Then, the tank was filled with high-purity argon. The main disc speed and planetary disc speed were set as 350 r/min and 700 r/min, respectively. To prevent the machine from overheating, each milling time was set at 50 min with a 10 min pause. The TiN-TiB_2_ nanocrystalline powders were obtained via 20 h effective ball-milling. After that, the second mechanical alloying process was used to prepare SiBCN-Ti powders. The c-Si (99.9% purity, 9.0 µm, China New Metal Materials Technology Co., Ltd., Beijing, China), h-BN (99.0% purity, 0.6 µm, Advanced Technology & Materials Co., Ltd., Beijing, China), graphite (99.5% purity, 8.7 µm, Qingdao Huatai Lubricant Sealing S&T Co., Ltd., Pingdu, China) powders, and the as-prepared TiN-TiB_2_ nanocrystalline powders were put into the ball milling tank filled with argon. The molar ratio of c-Si: h-BN: graphite was 2:1:3, and the mass fraction of TiN-TiB_2_ nanocrystals in the mixed powders was selected to 5 wt%, 10 wt%, 15 wt%, 20 wt%, and 30 wt%. Then, the SiBCN-Ti powders with different Ti contents were obtained by high-energy ball-milling, and the basic parameters of this ball-milling procedure were the same as the first mechanical alloying process.

### 2.2. Preparation of SiBCN-Ti Series Ceramics

The SiBCN-Ti series ceramics with different Ti contents were fabricated by hot-pressing. The as-milled SiBCN-Ti ceramic powders were transferred into a graphite mold with a diameter of 36 mm. The sintering process was conducted at 1900 °C for 30 min under flowing nitrogen with a pressure of 60 MPa. A heating rate of 30 °C/min was applied when the sintering temperature was lower than 1200 °C, and the rate was reduced to 25 °C/min when the temperature was higher than 1200 °C. Axial pressure was applied once the temperature reached 1200 °C, and the pressure was released completely when the temperature decreased to 1200 °C. After sintering, the furnace was allowed to cool naturally. The as-sintered samples with the thickness ~10 mm prepared from the above SiBCN-Ti powders with 5 wt%, 10 wt%, 15 wt%, 20 wt%, and 30 wt% Ti contents were labeled as Ti-5, Ti-10, Ti-15, Ti-20, and Ti-30, respectively.

### 2.3. Oxidation Experiments

The SiBCN-Ti series ceramics were processed into 3 × 4 × 5 mm pieces and subjected to oxidation experiments in a static air atmosphere. The oxidation temperature was 1500 °C, and the oxidation time was set as 1, 2, 4, 6, 8, and 12 h. Then, the samples underwent a natural cooling process to reach room temperature.

### 2.4. Characterization

The phase composition of the oxide layer on SiBCN-Ti series ceramics was investigated using an X′PERT Cu Kα-ray X-ray diffractometer (XRD) from Panalytical, Almelo, The Netherlands. The scanning speed was 10°/min, and the scanning range was 10–90°. An inVia-Reflex Raman spectrometer produced by Renishaw (Wotton-under-Edge, UK) was used to measure the Raman spectrum of the oxide layer. The thermal analysis of the ceramics was investigated under an air atmosphere with a heating rate of 10 °C/min using the TGA/DSC3^+^ synchronous thermal analyzer from Mettler, Columbia, MD, USA. A NanoLab 600i scanning electron microscope (SEM) from FEI (Hillsboro, OR, USA) was used to observe the surface and interfacial morphology of the oxidized SiBCN-Ti ceramics. The microstructure and elemental distribution at the interface between the oxide layer and the ceramics were analyzed using a Talos F200x transmission electron microscope (TEM) produced by FEI, and the samples for microscopic observation were prepared using a NanoLab 600i focused ion beam microscope (FIB) produced by FEI.

## 3. Results and Discussion

### 3.1. Evolution of Surface Composition during Oxidation

**Figure 1:**
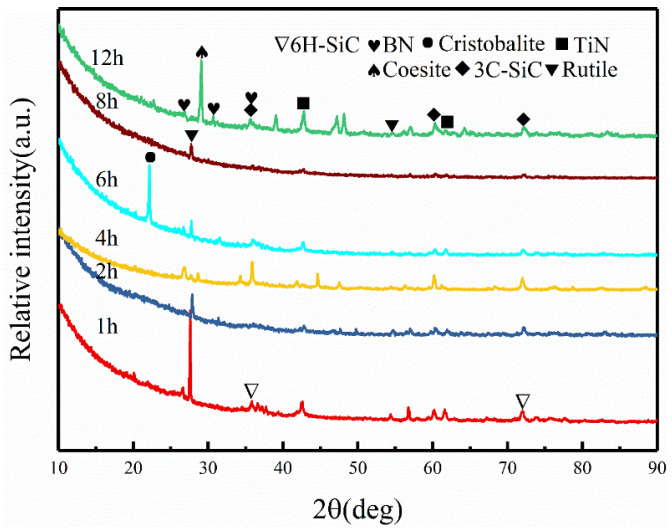
XRD patterns of the Ti-5 ceramics oxidized at 1500 °C at different times.

Figure 1 displays the surface phase composition of Ti-5 ceramics after oxidation at 1500 °C for 1–12 h. The oxidation behavior of SiBCN-Ti series ceramics varies with oxidation time. When the oxidation time is short, such as 1 or 2 h, the surface of the SiBCN-Ti series ceramic is composed of rutile, coesite, 3C-SiC, and TiN. With the oxidation time increasing to 4–12 h, the peak intensities of 6H-SiC gradually decrease, and intensities of 3C-SiC increase, which indicates that the transformation from 6H-SiC to 3C-SiC occurs with the increase in the oxidation time at 1500 °C. Additionally, with the increase in oxidation time, the diffraction peaks of the rutile become less sharp, indicating that the rutile phase is distributed in the oxide layer in the form of nanocrystals. When the oxidation time is relatively shorter, the Ti(C, N) is not fully oxidized, and it mostly maintains its original form. When the oxidation time is longer than 4 h, the Ti(C, N) is mostly in the form of TiN. Nevertheless, the SiO_2_ transforms into the cristobalite phase at an oxidation time of 6 h, and it is interesting that the SiO_2_ reconverts to the coesite phase when the oxidation time reaches 8 h. When the oxidation time reaches 12 h, the intensity of the rutile diffraction peaks relative to that of the coesite diffraction peaks decreases significantly. There are two possible reasons for the above phenomenon. Firstly, on the surface of the oxide layer, more and more SiO_2_ covers the TiO_2,_ resulting in the non-detection of the TiO_2_ diffraction peaks. Secondly, some of the TiO_2_ reacts with SiO_2_ to form silicates, thus decreasing the intensities of TiO_2_ peaks.

**Figure 2:**
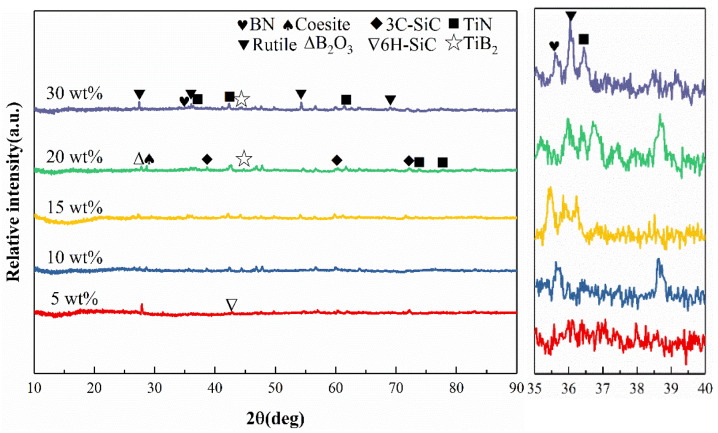
XRD patterns of the hot-pressed various SiBCN-Ti ceramics oxidized at 1500 °C for 2 h.

Figure 2 shows the surface XRD patterns of SiBCN-Ti series ceramics with different Ti contents after oxidation at 1500 °C for 2 h. With the Ti content increasing from 5 to 30 wt%, the diffraction peaks of 3C-SiC decrease, and those of coesite increase gradually. This is mainly due to the fact that more gases (CO, CO_2_, NO, NO_2_, etc.) are generated, and then more pores are formed in the oxide layer during the oxidation of Ti(C, N), resulting in the formation of more pores, thus promoting the oxidation of 3C-SiC. With the extension of oxidation time, the diffraction peaks of BN become broader and shorter, indicating that BN is constantly oxidized, and the surviving BN in the oxide skin is possibly amorphous or nanocrystalline.

**Figure 3:**
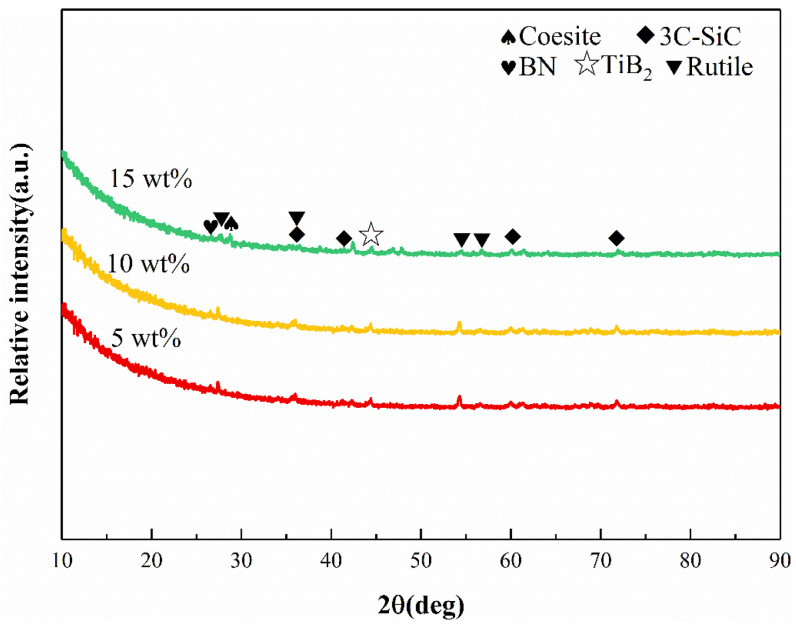
XRD patterns of the hot-pressed various SiBCN ceramics oxidized at 1500 °C for 4 h.

As shown in Figure 3, when the oxidation time is 4 h, the main phases in the oxide layer are coesite, 3C-SiC, rutile, and BN. The diffraction intensities of the rutile peaks are enhanced with the increase in Ti content, indicating that the higher Ti content is favorable for the formation of the rutile phase during the oxidation procedure. However, the large amount of rutile phase is not beneficial to the oxidation resistance of the SiBCN-Ti ceramics, so the Ti-5 can be chosen for the following characterizations.

**Figure 4:**
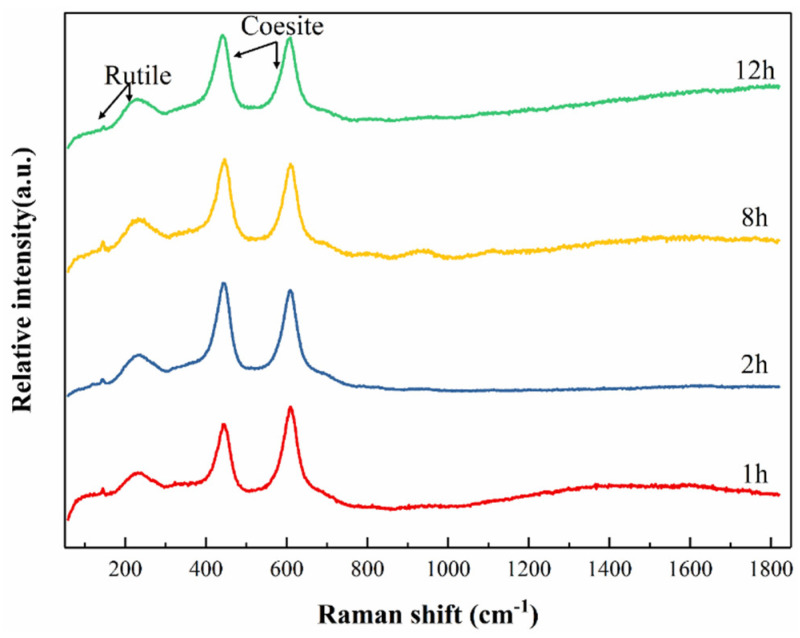
Raman spectra of the hot-pressed Ti-5 ceramics oxidized at 1500 °C for 1~12 h.

Figure 4 shows the Raman spectra of Ti-5 ceramics after oxidation at 1500 °C for 1, 2, 8, and 12 h. The broadened G bands in the range of 1000–1800 cm^−1^ belong to the graphite or h-BN, indicating that the h-BN in the oxide layer of Ti-5 ceramics shows an amorphous or nanocrystalline state. However, the D band generated by the sp^2^ hybridization of carbon is not detected in all samples, demonstrating the absence of ordered carbon on the oxide surface of Ti-5 ceramics. In addition, rutile vibrational peaks can be observed at ~144.22 and ~231.38 cm^−1^, and the positions of these peaks are closely related to the vibration of the Ti–O bond and O–Ti–O bond in rutile crystals. The peaks of coesite were observed at ~442.30 and ~609.26 cm^−1^, and the two peaks corresponded to the bending vibration of Si-O-Si and the stretching vibration of Si-O, respectively. Interestingly, when the oxidation time reaches 12 h, the vibrational peak of rutile at 144.22 cm^−1^ becomes weaker, and the vibrational peak of coesite at 442.30 cm^−1^ becomes relatively stronger. After a long time of oxidation, the vibration of the Si–O bond on the surface of the oxide layer is greater than that of the Ti–O bond, resulting in an enhanced coesite peak and a weakened rutile peak. Nevertheless, Ti-5 ceramics only have a broadened peak in the range of 200–300 cm^−1^, indicating the amorphous nature of Ti and O atoms. These results are consistent with the absence of the TiO_2_ diffraction peaks in the above XRD spectra.

**Figure 5:**
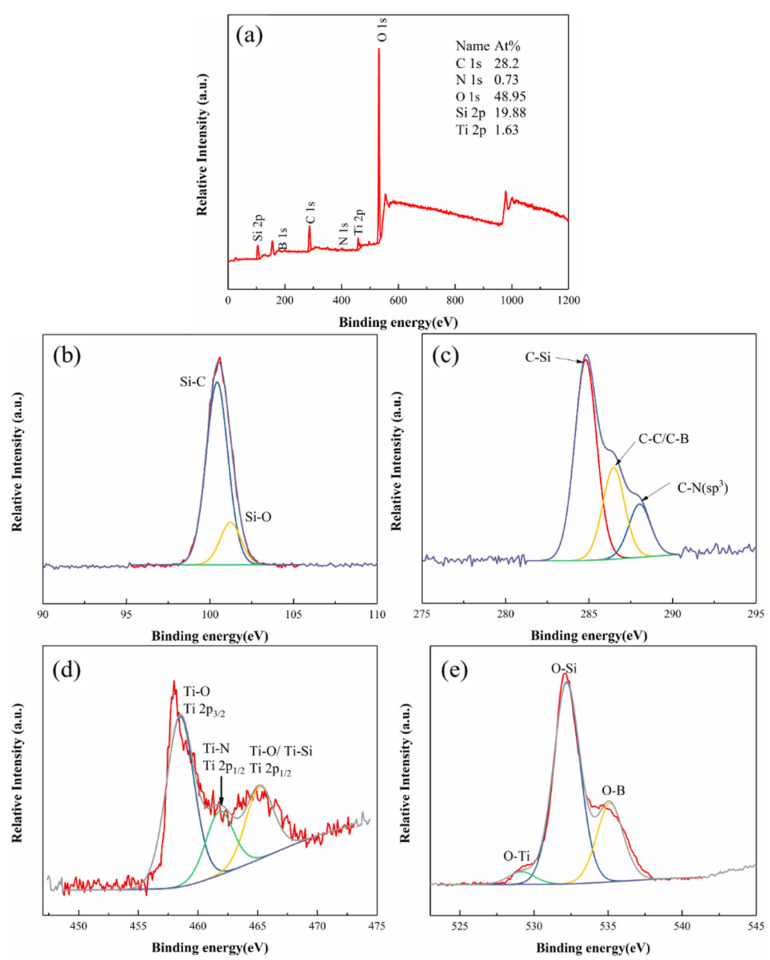
XPS spectra of the hot-pressed Ti-5 ceramics oxidized at 1500 °C for 1 h: (**a**) Survey; (**b**) Si 2p; (**c**) C 1s; (**d**) Ti 2p; (**e**) O 1s.

Figure 5 shows the XPS spectrum of Ti-5 ceramics oxidized at 1500 °C for 1 h. From Figure 5a, it can be seen that the B and N elements on the surface of the oxidized SiBCN-Ti series ceramics basically disappear. Meanwhile, the surface of the sample is dominated by the O and Si elements, with a small amount of Ti element. As shown in Figure 5b, it is known that the Si–C bond on the sample surface transforms into a Si–O bond during the oxidation procedure. From Figure 5c, it can be seen that the C element exists mainly as a C–Si bond, with a small amount of incompletely oxidized C–C and C–N bonds. In Figure 5d, Ti-C and Ti-N are converted to Ti-O after oxidation. From Figure 5e, the O element exists mainly as an O–Si bond, and the O–B and O–Ti bonds with a relatively lower intensity can also be observed.

### 3.2. Thermal Stability during Oxidation

**Figure 6:**
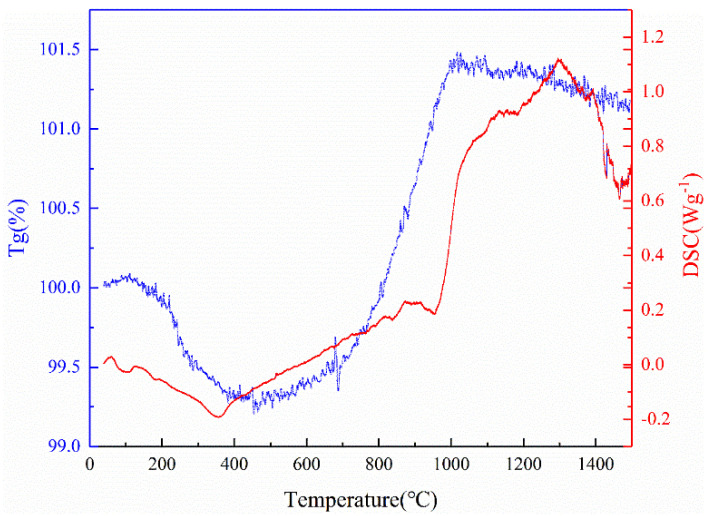
TG-DSC curves of the hot-pressed Ti-5 ceramics heated to 1500 °C in air.

Figure 6 shows the TG and DSC curves of Ti-5 ceramics in the air atmosphere. From room temperature to 443.26 °C, the ceramics mainly undergo low-temperature oxidation reaction, and an obvious exothermic peak at ~350 °C can be observed. During this process, volatile gases such as CO_2_, CO, and NO_x_ are produced, and the mass of the ceramics decreases gradually. At 443.26 °C, the mass loss of Ti-5 is only 0.71 wt%. However, when the temperature ranges from 443.26–1000.06 °C, the mass of the sample shows an obvious increment, and another apparent exothermic peak at ~960 °C can be detected due to the formation of oxides such as B_2_O_3_ and SiO_2_. Whereas when the temperature is higher than 1000.06 °C, the mass loss of the ceramics occurs again, accompanied by a clear endothermic peak at ~1300 °C, which is caused by the further oxidation of SiBCN-Ti ceramics under the oxide layer.

### 3.3. Microstructural Evolution of the Oxide Layer

**Figure 7:**
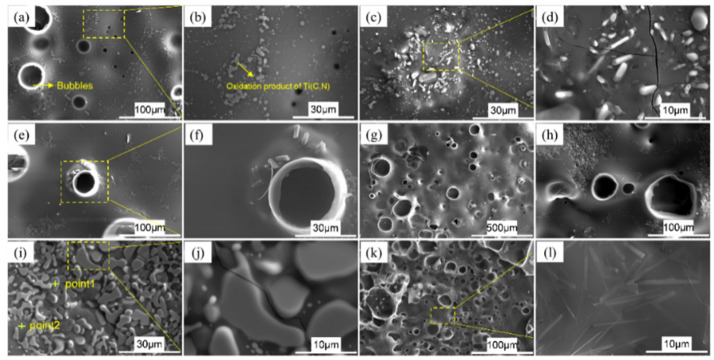
The surface morphologies of hot-pressed Ti-5 ceramics after oxidation at 1500 °C: (**a**–**d**) 1 h; (**e**,**f**) 2 h; (**g**–**j**) 8 h; (**k**,**l**) 12 h.

**Figure 8:**
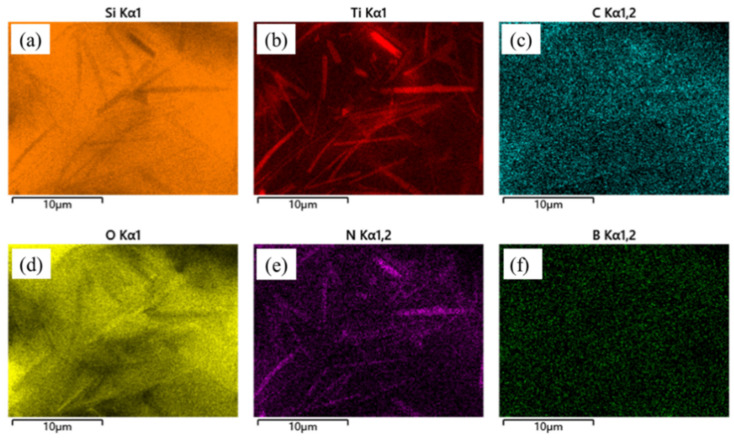
SEM image and element distribution of hot-pressed Ti-5 ceramics after oxidation at 1500 °C for 12 h: (**a**) Si mapping; (**b**) Ti mapping; (**c**) C mapping; (**d**) O mapping; (**e**) N mapping; (**f**) B mapping.

In order to study the oxidation process of SiBCN-Ti series ceramics, the microstructure evolution of the Ti-5 oxide layer at 1500 °C for different oxidation times was investigated. Figure 7 shows the surface morphologies of Ti-5; it can be observed that some bubbles are formed on the surface of ceramics after a 1 h oxidation. This phenomenon is primarily attributed to the formation and evolution of volatile gases, including CO, CO_2_, NO, and B_2_O_3_, which are produced by the oxidation of BN(C). As the oxidation reaction progresses, the amounts of gases increase gradually. Ultimately, the oxide layer transforms to a loose and porous structure after a 12 h oxidation. Meanwhile, some light color precipitates were also found on the oxide layer. As shown in Figure 7g–j, when the oxidation time reaches 8 h, in addition to the increase in pores, the accumulation of a light-gray fusiform structure is also observed on the surface of the oxide layer. As shown in Figure 7i, the elemental analysis was performed on the oxidized surface with and without a light-gray fusiform structure, marked as point 1 and point 2, respectively. The EDS result shows that Ti, N, and O are the main elements in point 1, indicating the existence of TiN and TiO_2_. Si and O are the main elements in point 2, proving the formation of SiO_2_. In Figure 7k,l, it can be observed that a multitude of elongated grain structures are uniformly dispersed on the oxide layer surface. Elemental surface scanning images of Ti-5 ceramics after 12 h oxidation are summarized in Figure 8; the disappearance of the C component indicates that the Ti(C, N) is oxidated completely, and these elongated grains, which exhibit a distinct crystalline structure, can be determined as TiN, which is also confirmed with the above XRD results.

**Figure 9:**
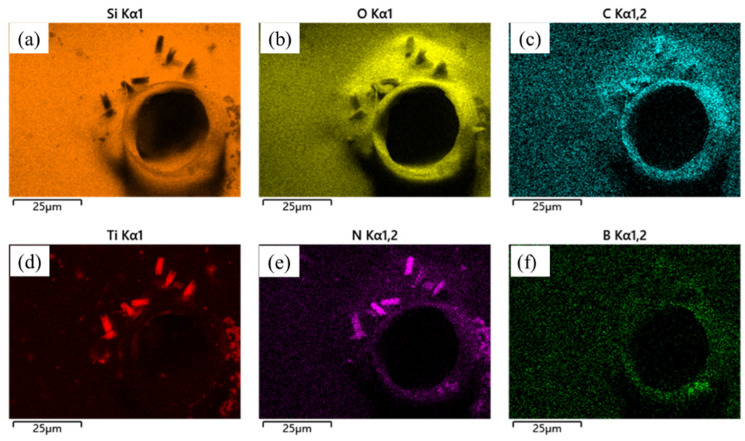
Element distribution of the hot-pressed Ti-5 ceramics after oxidation at 1500 °C for 2 h: (**a**) Si mapping; (**b**) O mapping; (**c**) C mapping; (**d**) Ti mapping; (**e**) N mapping; (**f**) B mapping.

As can be seen from Figure 9, the O, B, and C elements are aggregated around the pores on the oxide layer due to the oxidation of BN(C). The short rod-shaped Ti(C, N) particles have grown at 1500 °C, but the particles have not been completely oxidized. It can be seen from Figure 9c,e that some of the long rods do not overlap with the C and N elements due to the oxidation of some C elements in the Ti(C, N), consistent with the results of the XRD analyses.

**Figure 10:**
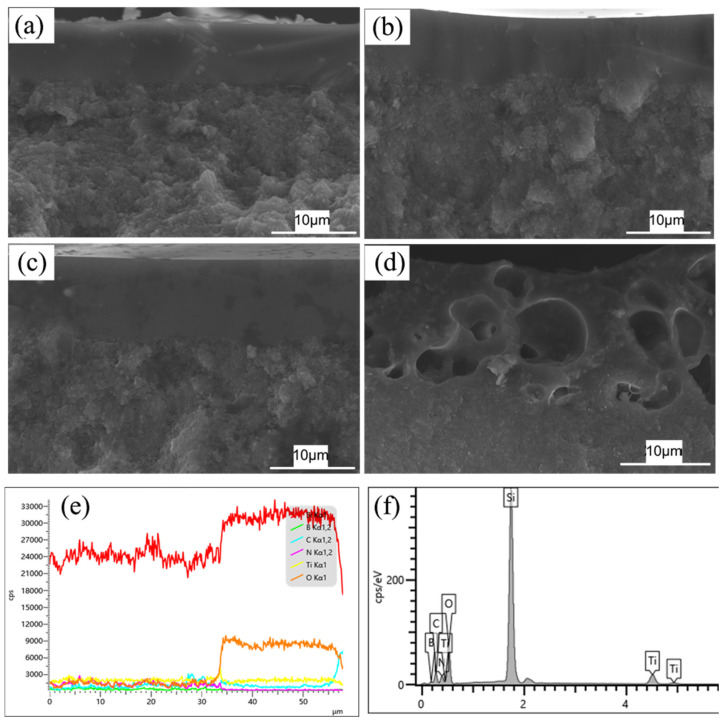
Cross-sectional morphologies and element distribution of the hot-pressed Ti-5 ceramics oxidized at 1500 °C: (**a**) 1 h; (**b**) 2 h; (**c**) 8 h; (**d**) 12 h; (**e**) EDS line scanning of the cross-section area from the lower ceramics to the upper oxide layer in Figure 10d; (**f**) EDS spot analysis of the oxide layer in Figure 10d.

Figure 10a–d depicts the microstructure and elemental composition of Ti-5 ceramics after undergoing oxidation at 1500 °C at different times. When the oxidation time is less than 8 h, the ceramics exhibit comparatively dense oxide layers. Conversely, after 12 h oxidation, the porous and less dense oxide layer can be observed. A comparative elemental analysis of the cross-sectional elements is shown in Figure 10e,f. The Si and O elements have significantly higher concentrations in the oxide layers compared to their respective concentrations in the base ceramics. Furthermore, this analysis also indicates the presence of Ti element in the oxide layer and base ceramics.

**Figure 11:**
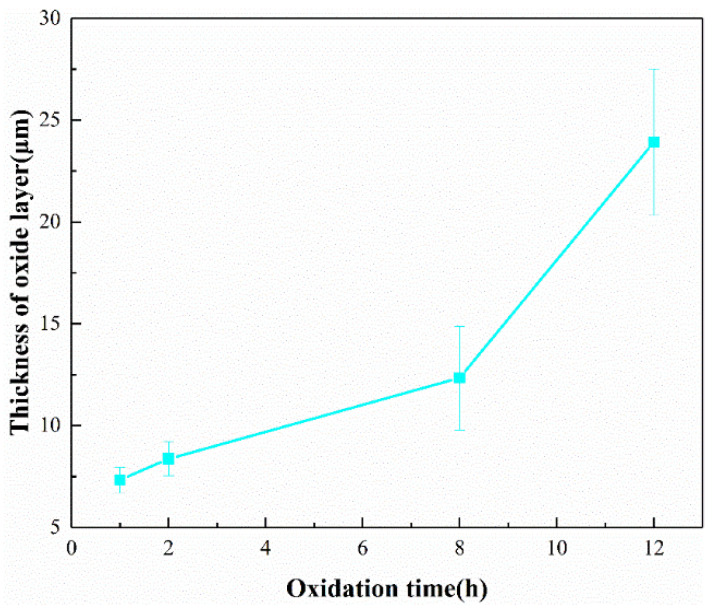
Oxide layer thickness as a function of oxidation time for the hot-pressed Ti-5 ceramics oxidized at 1500 °C.

Figure 11 illustrates the thickness of the oxide layers of Ti-5 ceramics after oxidation at 1500 °C at different times. As the oxidation time increases, the thickness of the oxide layer of Ti-5 ceramics increases, and it can be observed that at an oxidation time of 5 h, the thickness of the oxide layer is ~10 μm. According to the previous study, the oxide layer thickness of the pure SiBCN ceramics after 5 h oxidation is ~30 μm [10]. Compared with the pure SiBCN ceramics, the oxide layer thickness of SiBCN-Ti ceramics shows a significant decrease, demonstrating that the incorporation of Ti is beneficial to enhancing the anti-oxidation properties of SiBCN ceramics.

**Figure 12:**
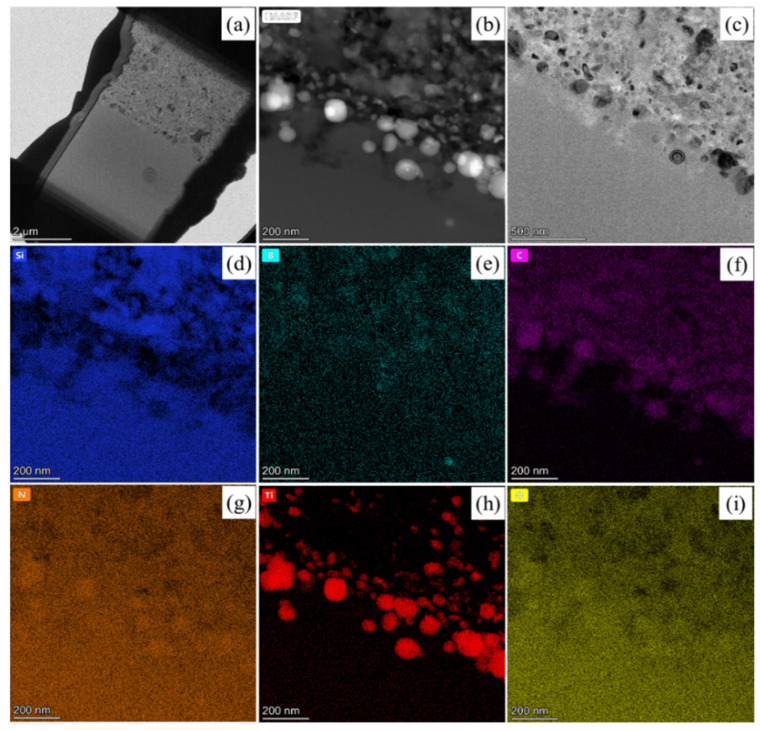
Oxide layer microstructure of the hot-pressed Ti-5 ceramics after oxidation at 1500 °C for 4 h: (**a**) A FIB slice; (**b**) HAADF-STEM image; (**c**) TEM bright-field image; (**d**–**i**) corresponding EDS maps.

In order to further investigate the microstructural evolution of the SiBCN-Ti series ceramics after oxidation, the interfacial microstructure between the oxide layer and the ceramics of the Ti-5 ceramics after oxidation at 1500 °C for 4 h was analyzed by TEM characterization (Figure 12). As depicted in Figure 12b,c, the oxide layer is predominantly constituted of an amorphous oxidation region and an incompletely oxidized region, and the latter region is predominantly composed of incompletely oxidized SiC, BN(C), and Ti(C, N). The elemental composition of the amorphous oxide phase is primarily constituted of Si, O, and Ti, which further proves that the primary component of the amorphous oxide region is rutile silicate glass, combined with the above XRD results. Figure 12d–i shows that the Si element is distributed in the ceramic substrate and the oxide layer, while the O element is mainly distributed in the oxide layer. The Ti element is mainly distributed in the Ti silicate glass, TiO_2_ nanocrystals, and incompletely oxidized Ti(C, N) nanocrystals, and the particle size of the above nanocrystals is ~200 nm. In addition, according to the elemental distribution of B, the surviving BN shows the nanocrystalline structure, and the particle size of the nanocrystals is tens of nanometers. These results are consistent with the conclusion from SEM and XRD characterization.

### 3.4. Thermodynamic Analysis of Possible Oxidation Reactions

**Figure 13:**
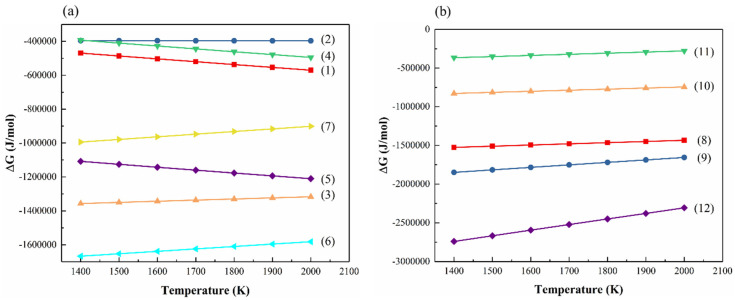
Calculated Gibbs free energy changes for the possible oxidation reactions (1)–(12) during oxidation tests.

The oxidation reactions of hot-pressed SiBCN-Ti ceramics during high-temperature oxidation are illustrated in Equations (1)–(12). As the thermodynamic data of BN(C) and Ti(C, N) remain unknown, their oxidation reactions are studied by calculating the reactions of BN, C, TiC, and TiN with O_2_. Furthermore, the Gibbs free energy changes in these reactions were calculated using the Factsage software (https://factsage.com/). The results are presented in Figure 13; it is evident that the Gibbs free energy changes in all the aforementioned reactions are negative. Therefore, all the reactions may potentially occur thermodynamically during the oxidation process. Furthermore, B_2_O_3_ has a melting point of 445 °C and a boiling point of 1500 °C will undergo vaporization when the oxidation temperature is 1500 °C, which is the primary reason for the formation of pores and bubbles in the oxide layer. However, the SiO_2_ and oxides of Ti(C, N) form the rutile and quartz composite oxide layer during oxidation, which is conducive to healing the pores and preventing the further oxidation of the ceramics. Consequently, the formation of cracks and the diffusion of oxygen along the cracks can be effectively prevented. Furthermore, in previous studies, the SiBCN series ceramics exhibit a cristobalite phase at high temperatures, which is unfavorable for the stability of SiBCN ceramics [23,30]. However, according to the above XRD results, the oxides of Ti(C, N) could inhibit the formation of the cristobalite phase and promote the generation of the coesite phase with more thermally stable characteristics; thus, the ceramics are able to maintain structural integrity at high temperatures. Therefore, the incorporation of Ti will enhance the oxidation resistance of the SiBCN series ceramics at high temperatures.

(1) $$2C\left(s\right)+O_{2}\left(g\right)=2CO\left(g\right)$$

(2) $$C\left(s\right)+O_{2}\left(g\right)=CO_{2}\left(g\right)$$

(3) $$4BN\left(s\right)+3O_{2}\left(g\right)=2B_{2}O_{3}\left(l\right)+2N_{2}\left(g\right)$$

(4) $$SiC\left(s\right)+O_{2}\left(g\right)=SiO\left(g\right)+CO\left(g\right)$$

(5) $$2SiC\left(s\right)+3O_{2}\left(g\right)=2SiO\left(g\right)+2CO_{2}\left(g\right)$$

(6) $$2SiC\left(s\right)+3O_{2}\left(g\right)=2SiO_{2}\left(g\right)+2CO\left(g\right)$$

(7) $$SiC\left(s\right)+2O_{2}\left(g\right)=SiO_{2}\left(l\right)+CO_{2}\left(g\right)$$

(8) $$2TiC\left(s\right)+3O_{2}\left(g\right)=2TiO_{2}\left(s\right)+2CO\left(g\right)$$

(9) $$TiC\left(s\right)+2O_{2}\left(g\right)=TiO_{2}\left(s\right)+CO_{2}\left(g\right)$$

(10) $$2TiN\left(s\right)+3O_{2}\left(g\right)=2TiO_{2}\left(s\right)+2NO\left(g\right)$$

(11) $$TiN\left(s\right)+2O_{2}\left(g\right)=TiO_{2}\left(s\right)+NO_{2}\left(g\right)$$

(12) $$2TiB_{2}\left(s\right)+5O_{2}\left(g\right)=2TiO_{2}\left(s\right)+2B_{2}O_{3}\left(l\right)$$

## 4. Conclusions

In this work, the oxidation resistance and microstructural evolution of the hot-pressed SiBCN-Ti ceramics with different Ti contents oxidized at 1500 °C for different times were studied, and the main conclusions are as follows:

(1) When the oxidation time is less than 4 h, the Ti(C, N) in SiBCN-Ti ceramics can maintain its original structure. When the oxidation time exceeds 4 h, the Ti(C, N) will transform mostly to TiN;
(2) The amorphous oxide layer is mainly composed of rutile silicate glass, which efficiently hinders the diffusion of oxygen;
(3) The introduction of Ti content is beneficial to the generation of the rutile coesite phase, which improves the anti-oxidation properties of the SiBCN-Ti series ceramics.

## Data Availability

The original contributions presented in the study are included in the article, further inquiries can be directed to the corresponding authors.
